# Supplementary material for: Retarding oxidation of copper nanoparticles without electrical isolation and the size dependence of work function
Source: Nat Commun. 2017 Dec 1;8:1894. doi: 10.1038/s41467-017-01735-6 (PMC5711799; doi:10.1038/s41467-017-01735-6)
Supplement: Supplementary file 1 — Supplementary Information [file 41467_2017_1735_MOESM1_ESM.pdf]

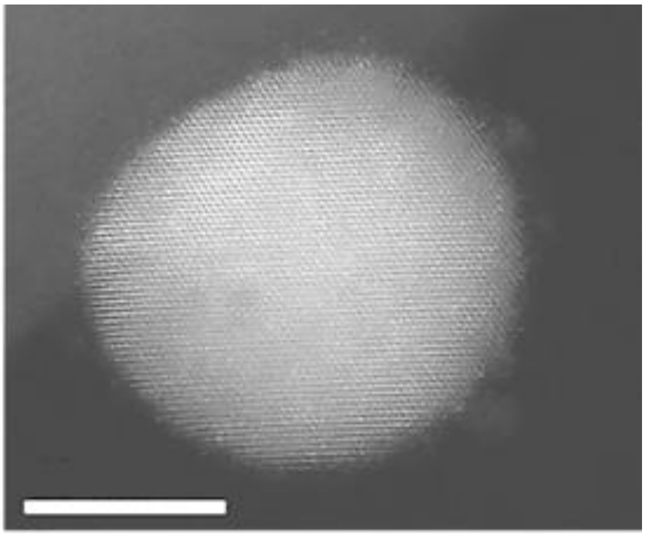

Supplementary Figure 1| Crystallinity of as-synthesised CuNPs

ADF-STEM image of a monocrystalline oleylamine capped CuNP. Scale bar 10 nm. The well-ordered crystalline lattice of Cu is clearly visible in the image.

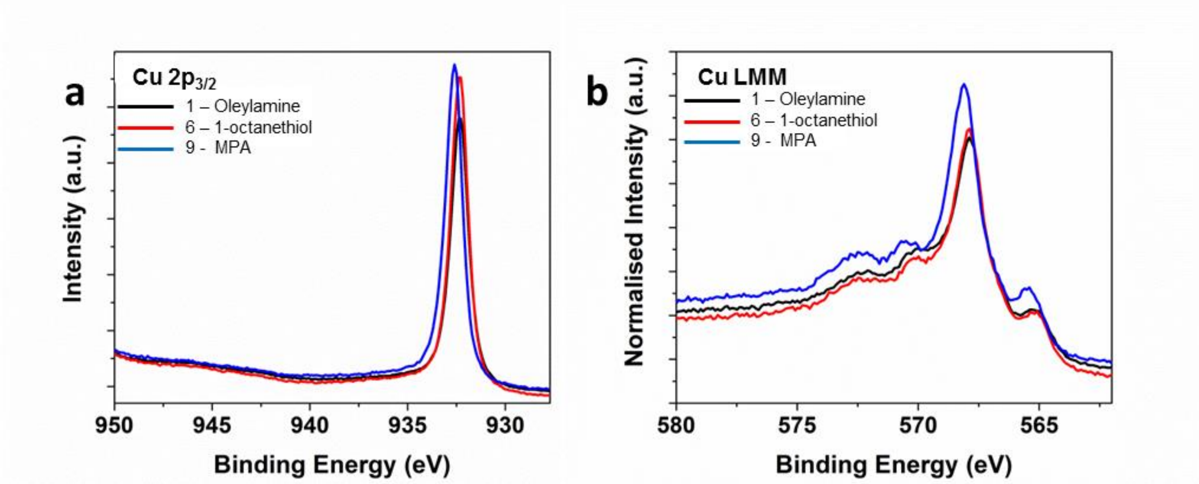

Supplementary Figure 2| XPS and Auger analysis of Cu NPs with ligands 1 6 and 9

XPS core level Cu $2p_{3/2}$ spectra **(a)** and Cu LMM Auger spectra **(b)** of oleylamine (1) capped Cu NPs, 1-octanethiol (6) capped Cu NPs and MPA (9) capped Cu NPs. The Cu $2p_{3/2}$ peak in (a) appears at 932.3 eV, 932.3 eV and 932.6 eV for 1, 6 and 9 respectively. From these spectra it is evident that no CuO is present in the samples since CuO would give a peak at 933.5 eV and a strong satellite peak at 943.6 eV[1]. The corresponding Cu LMM Auger spectra are given in (b) of this figure, because Auger peak energies can also be used to elucidate the

oxidation state of Cu. The Auger spectra have a similar shape for all three samples with binding energies of 567.9 eV, 567.9 eV and 568.1 eV for the most intense peak of 1, 6 and 9 respectively. If Cu_2O was present, there would be a peak at 570-570.4 eV and the shoulder peak at 565.2, 565.2 and 565.4 eV for 1, 6 and 9 would be surpressed[2]. The absence of peaks at ~570 eV and the presence of a sharp peak at 565.2-565.4 eV together show that the CuNPs are in the Cu^0 oxidation state, so the synthesis and/or ligand exchange processes have not resulted in oxide formation (although the possibility of an extremely thin layer of oxide species just below the resolution of the instrument cannot be ruled out entirely). Notably, the difference in binding energy with ligand type is not due to a variation of surface chemistry, since the Auger parameter is the same within error for all 3 ligands: 1851.0 eV ± 0.1 eV, 1851.0 eV ± 0.1 eV and 1851.1 eV ± 0.1 eV for 1, 6 and 9 respectively, and for all other ligands investigated in this study: see Supplementary Table 1. The apparent differences in binding energy are attributed to an instrumental effect, since the measurements were made on consecutive days. This is confirmed by the data presented in Supplementary Figure 3, which shows data for samples prepared in the same way but analysed sequentially on the same day, for which there are no shifts in binding energy.

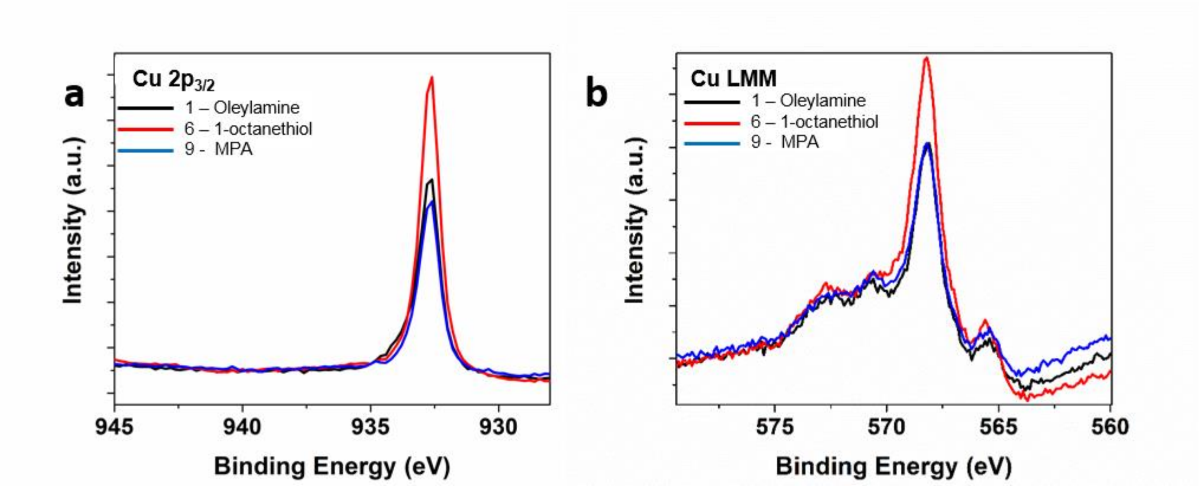

Supplementary Figure 3| Repeated XPS and Auger study of Cu NPs with ligands 1 6 and 9

XPS core level Cu $2p_{3/2}$ spectra **(a)** and Cu LMM Auger spectra **(b)** of fresh oleylamine (1) capped CuNPs, 1-octanethiol (6) coated CuNPs and MPA (9) coated CuNPs. The samples were prepared in the same way as those in Supplementary Figure 2, although analysed sequentially on the same day. The Auger parameters are the same within experimental error:

1851.2 eV ± 0.1 eV, 1851.1 eV ± 0.1 eV and 1851.1 eV ± 0.1 eV for samples 1, 6 and 9 respectively.

Supplementary Table 1| Auger parameters of oleylamine and thiol capped CuNPs

The similar Auger parameters for all of the thiolate ligands investigated indicate that the binding chemistry of the ligands to the CuNPs is not strongly dependent on the tail group.

	Ligand	Auger parameter ± 0.1 (eV)
1	Oleylamine	1851.1
2	1-octadecanethiol	1851.2
3	2-{2-[2-(2-mercaptoethoxy)ethoxy]ethoxy}ethanol	1851.2
4	1-Decanethiol	1851.0
5	6-mercaptohexanoic acid	1851.2
6	1-Octanethiol	1851.1
7	2-(2-methoxyethoxy)ethanethiol	1851.2
8	1,2-pentanedithiol	1851.1
9	MPA	1851.1

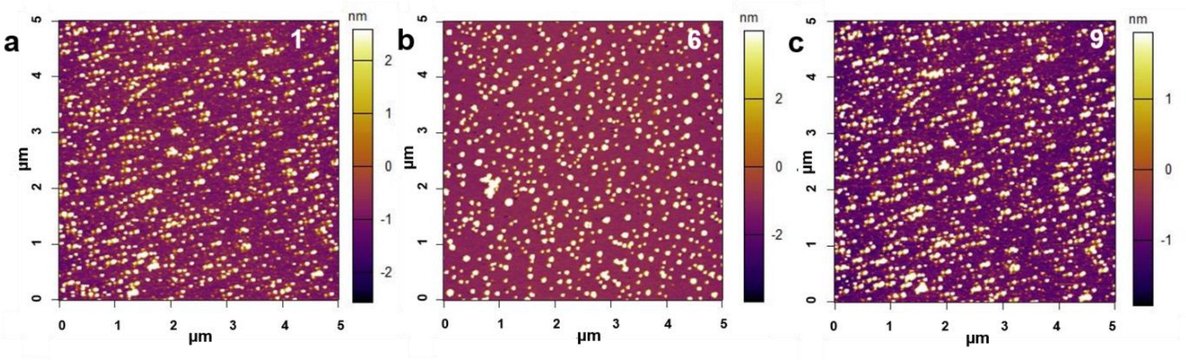

Supplementary Figure 4| AFM analysis of tethered CuNPs before and after ligand exchange

(a), **(b)** and **(c)** are representative AFM images of 1, 6 and 9 capped CuNPs used for the particle size analysis given in Fig. 3 of the manuscript. The area imaged is 5×5 µm and the samples were supported on a silicon wafer.

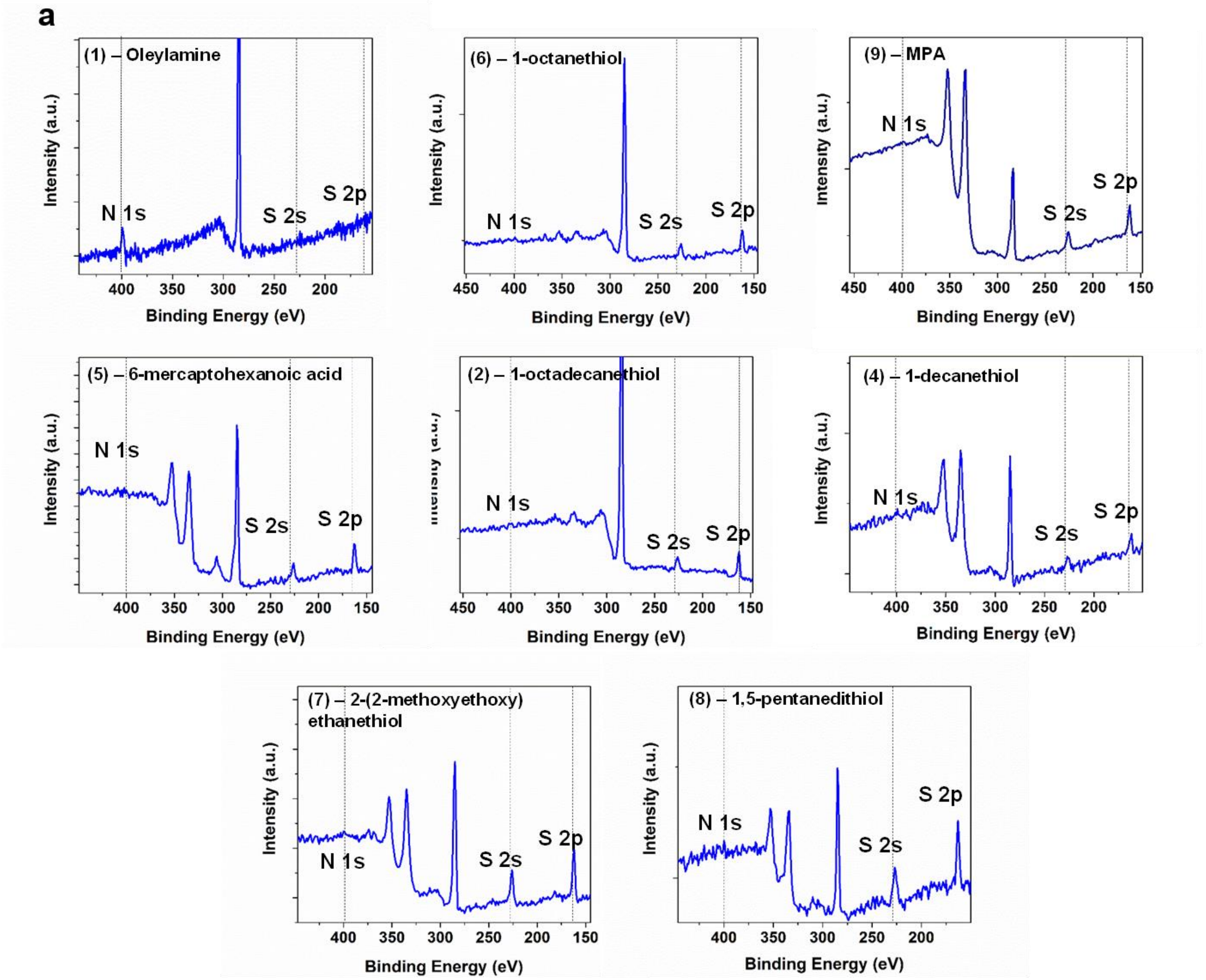
a
(1) – Oleylamine
(6) – 1-octanethiol
(9) – MPA
(5) – 6-mercaptohexanoic acid
(2) – 1-octadecanethiol
(4) – 1-decanethiol
(7) – 2-(2-methoxyethoxy) ethanethiol
(8) – 1,5-pentanedithiol
N 1s
S 2s
S 2p
Intensity (a.u.)
Binding Energy (eV)

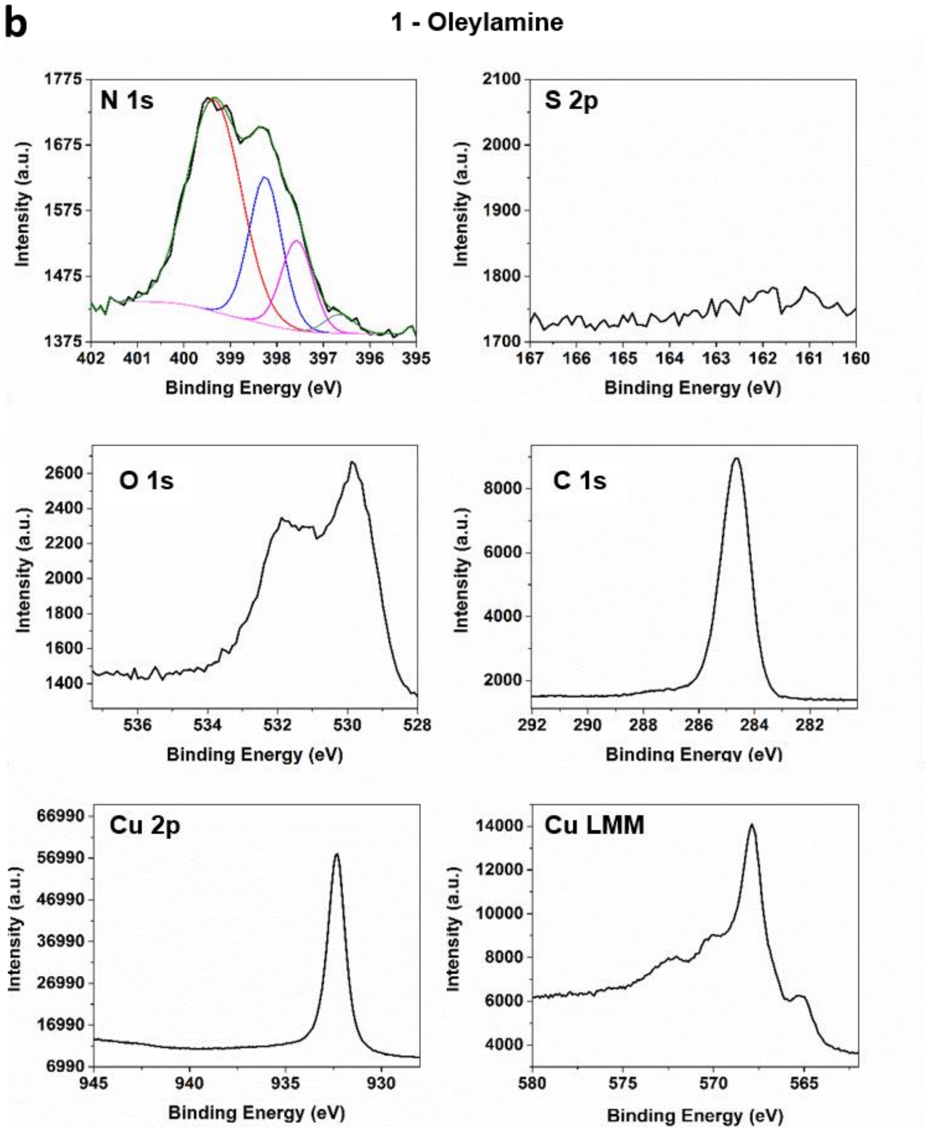
b
1 - Oleylamine
N 1s
Intensity (a.u.)
Binding Energy (eV)
S 2p
O 1s
C 1s
Cu 2p
Cu LMM

c 6 – 1-octanethiol

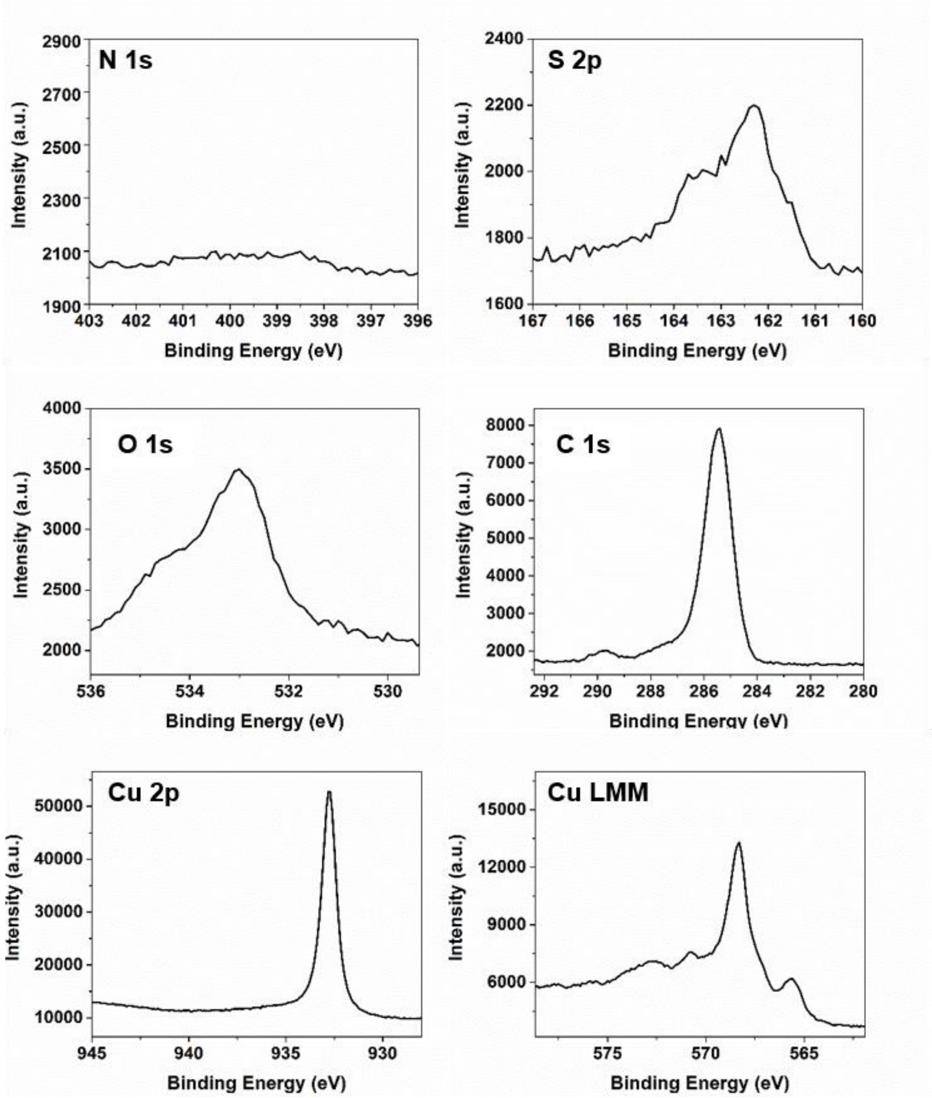

d

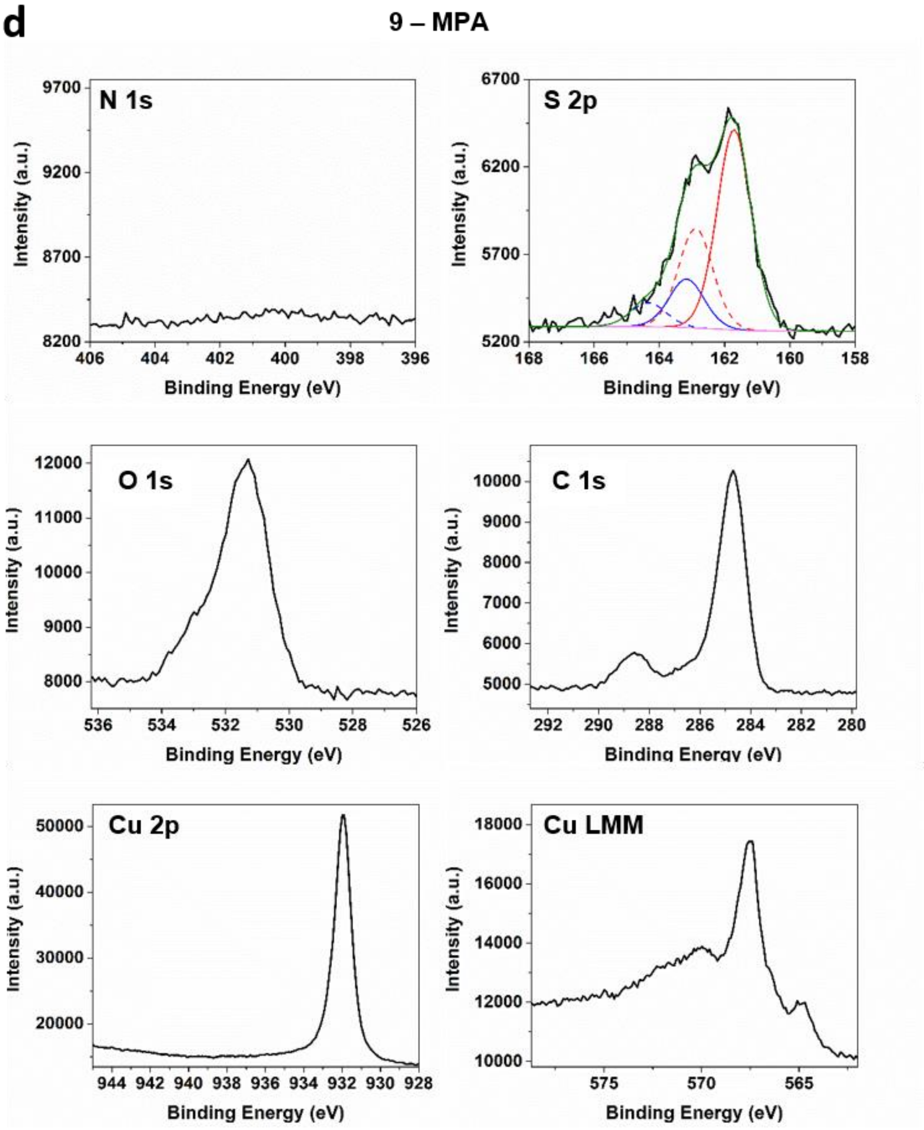

9 – MPA
N 1s
S 2p
O 1s
C 1s
Cu 2p
Cu LMM
Intensity (a.u.)
Binding Energy (eV)

e **5 – 6-mercaptohexanoic acid**

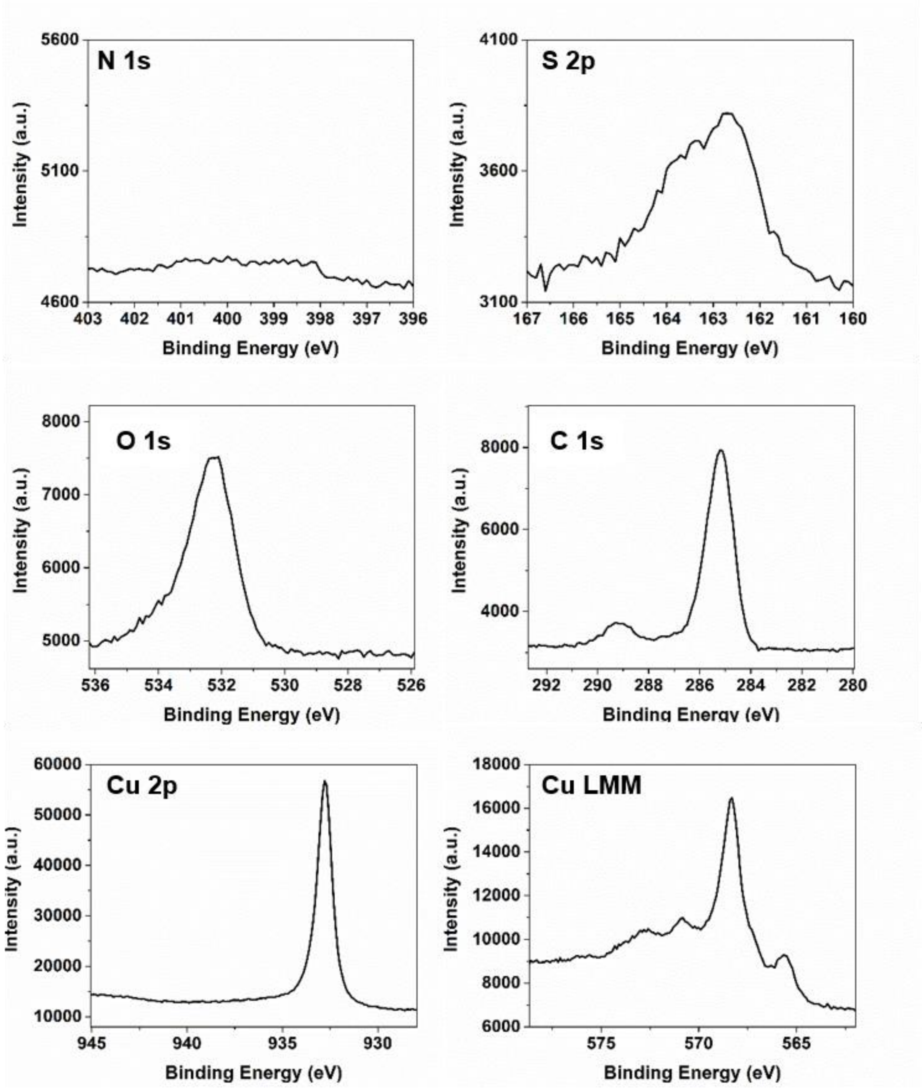

f

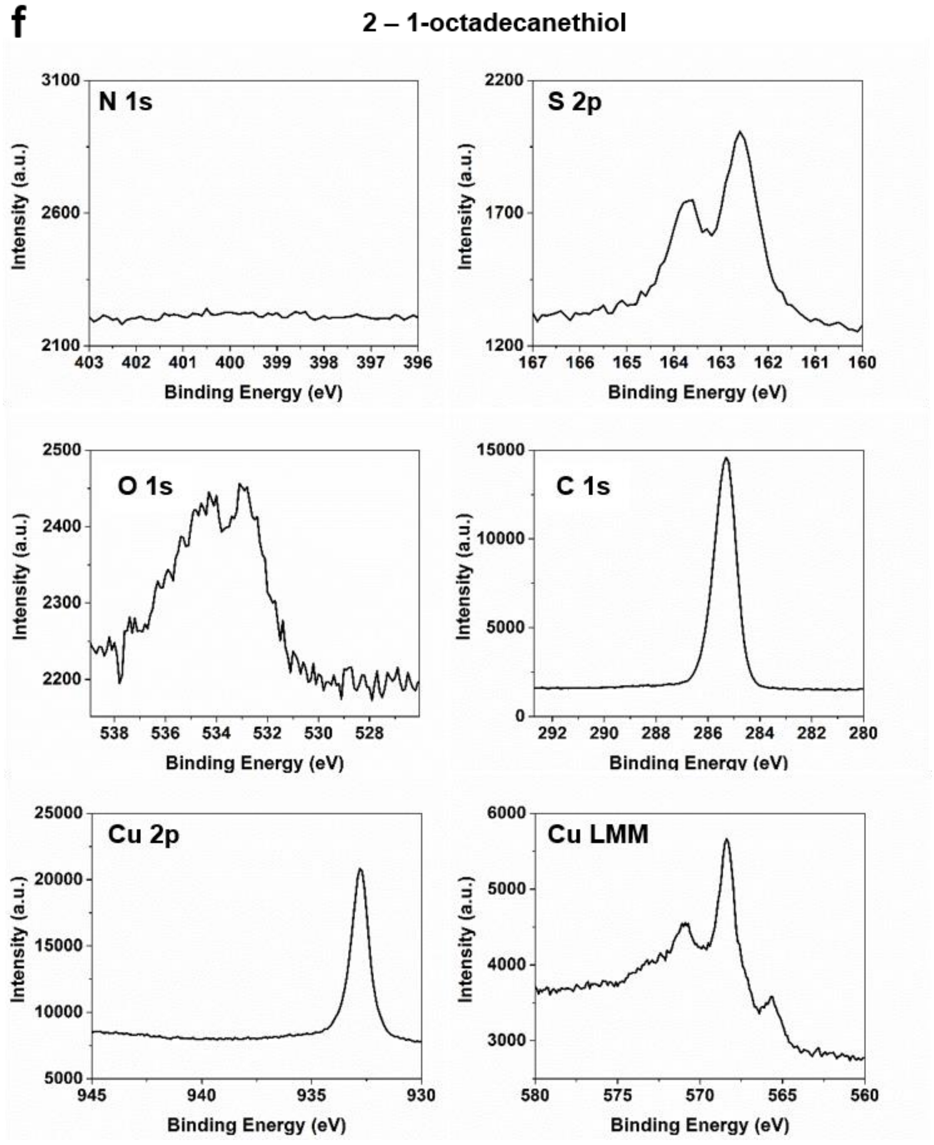

2 – 1-octadecanethiol
N 1s
S 2p
O 1s
C 1s
Cu 2p
Cu LMM
Intensity (a.u.)
Binding Energy (eV)

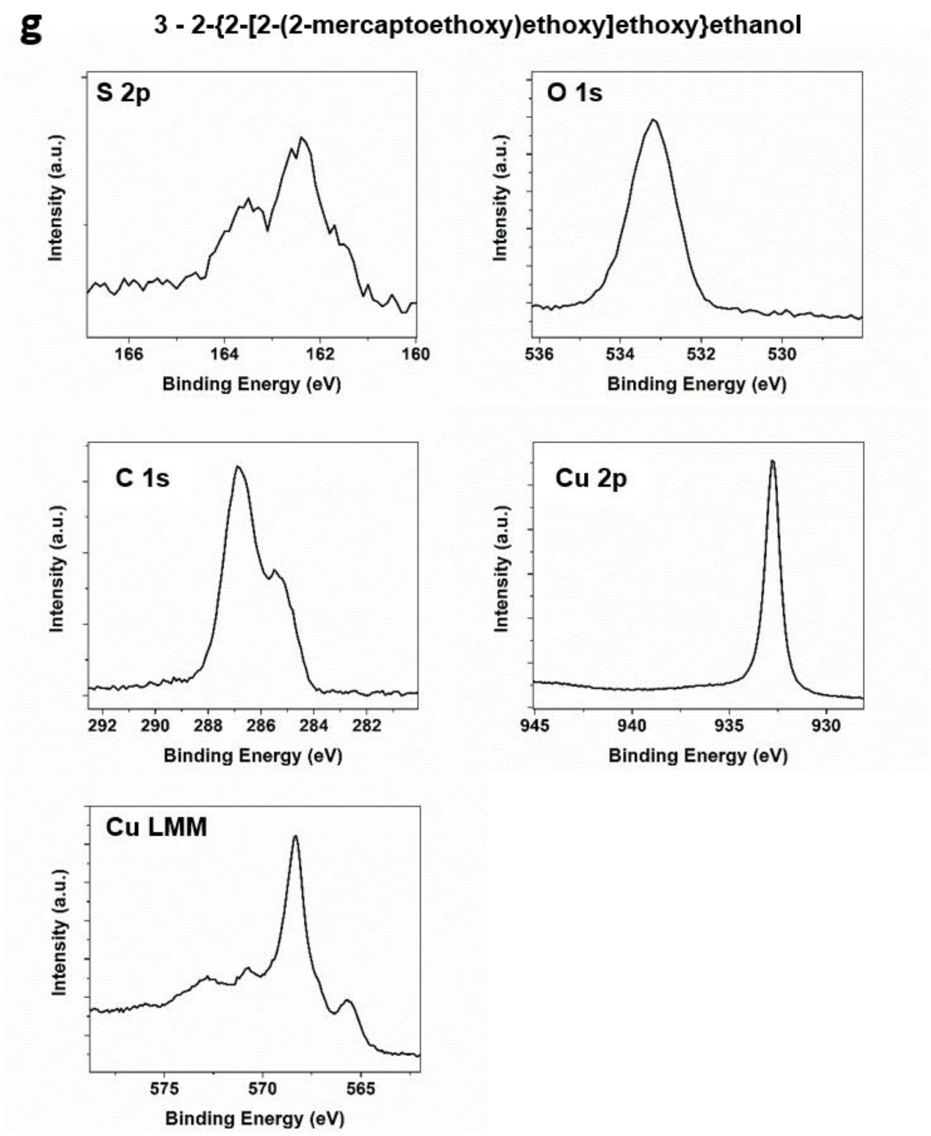
g
3 - 2-{2-[2-(2-mercaptoethoxy)ethoxy]ethoxy}ethanol
S 2p
Intensity (a.u.)
166
164
162
160
Binding Energy (eV)
O 1s
Intensity (a.u.)
536
534
532
530
Binding Energy (eV)
C 1s
Intensity (a.u.)
292
290
288
286
284
282
Binding Energy (eV)
Cu 2p
Intensity (a.u.)
945
940
935
930
Binding Energy (eV)
Cu LMM
Intensity (a.u.)
575
570
565
Binding Energy (eV)

h **4 – 1-decanethiol**

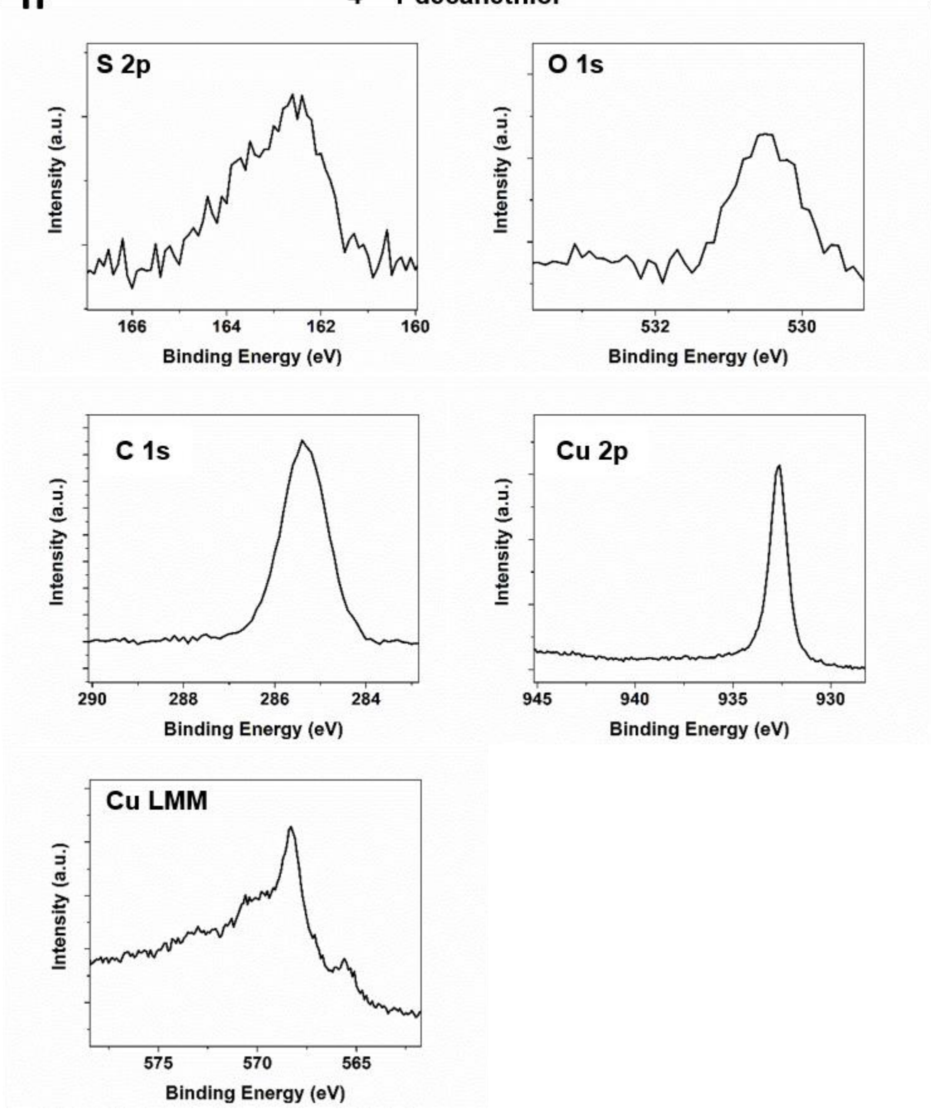

i **7 - 2-(2-methoxyethoxy)ethanethiol**

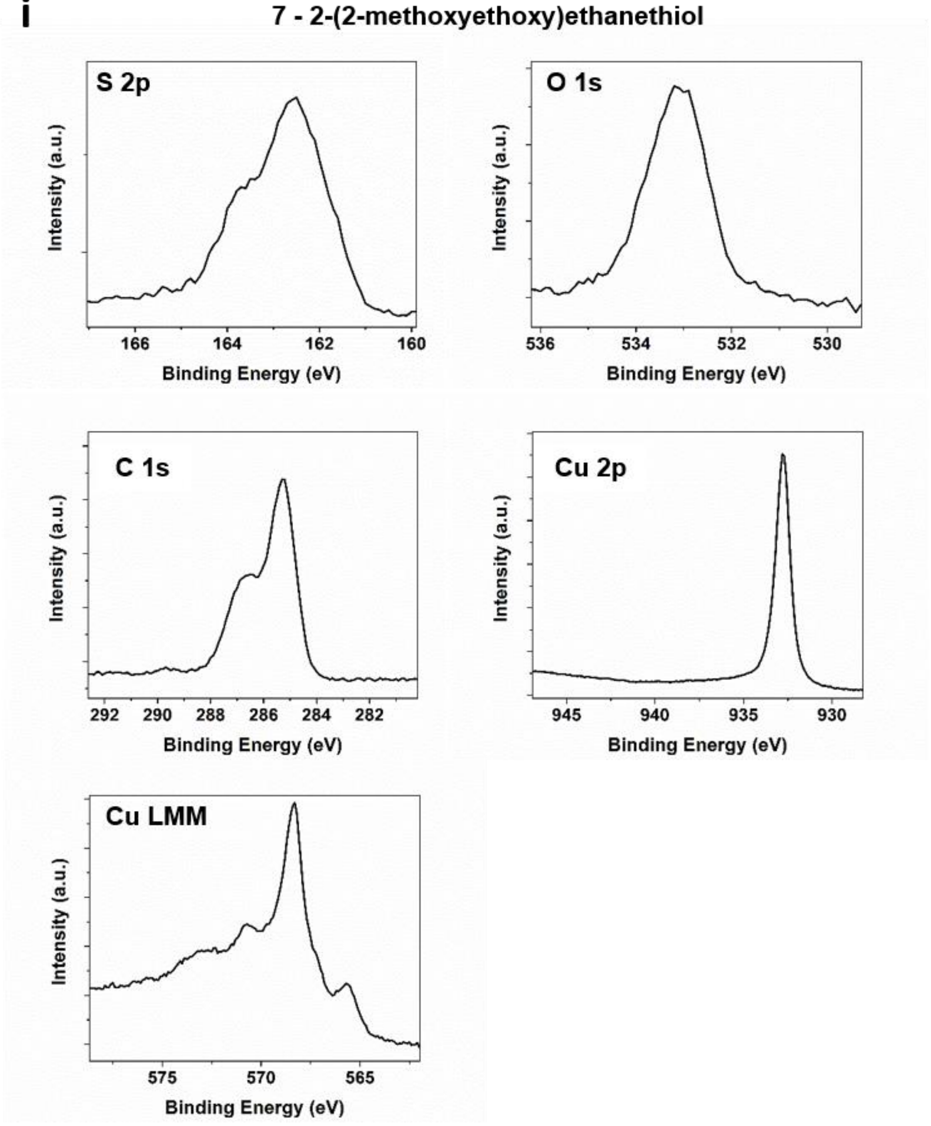

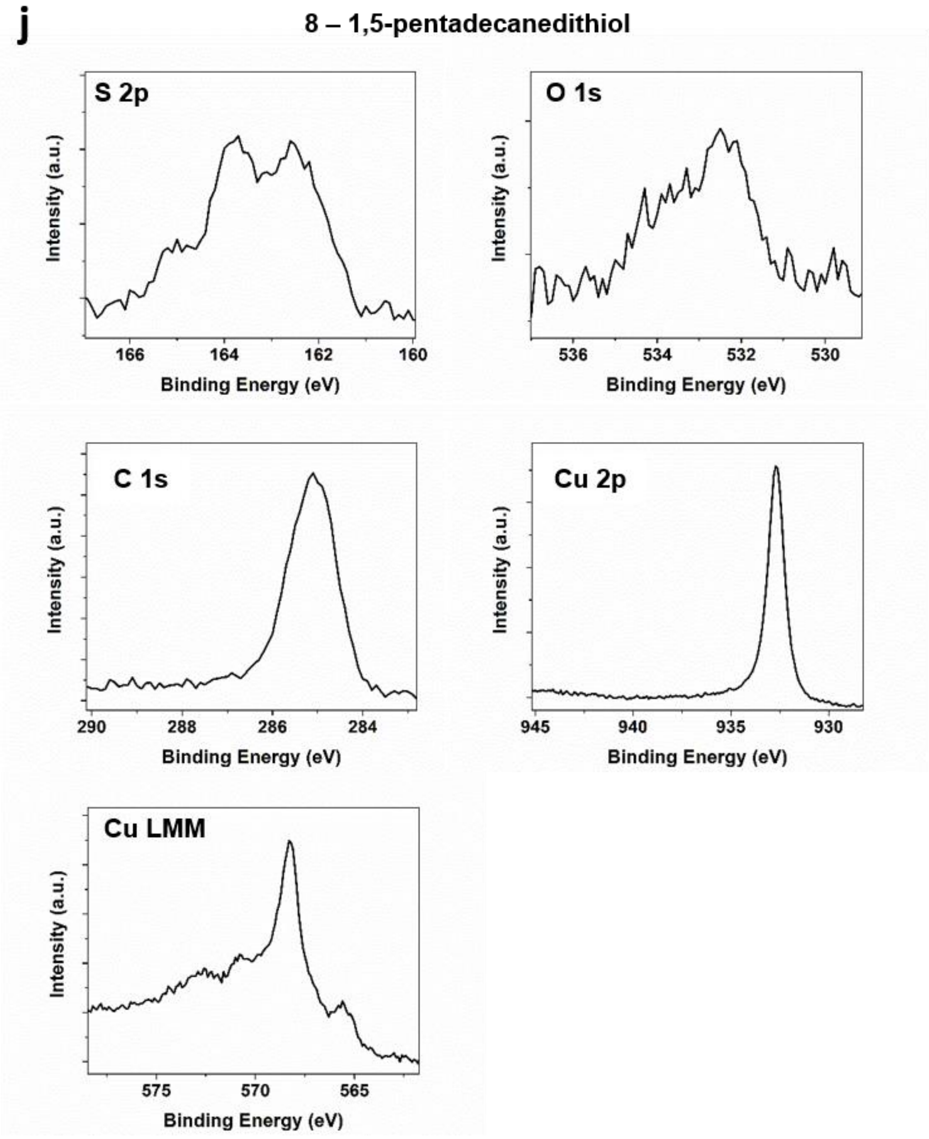

Supplementary Figure 5| XPS and Auger Cu LMM analysis of CuNPs capped with ligands (1)-(9)

Data sets given in order in which referred to in main manuscript. **(a)** Survey scans for all 8 thiol ligands show the disappearance of the N1s peak and the appearance of S2s and S2p peaks when oleylamine (1) is exchange with all 8 thiol ligands. **(b)** In the high resolution N1s region for oleylamine capped CuNPs (1) the peaks at 399.3 eV and 398.3 eV are assigned to physisorbed and chemisorbed oleylamine respectively[3]. As expected, no S is present in (1). **(c-j)** High resolution XPS spectra for all thiol ligands. The N1s regions for ligands (2), (5), (6) and (9) show no detectable N, which confirms the efficiency of ligand exchange. For (9) the peaks at ~161.7 eV and ~162.9 eV are assigned to $S2p_{3/2}$ and $S2p_{1/2}$ doublet with an

intensity ratio of 2:1 corresponding to the thiolate species[4]. The doublet at ~163.2 eV and ~164.4 eV are due to physisorbed/unbound MPA ligands[4].

Supplementary Table 2| AFM particle size analysis using different AFM tips

Particle size analysis (mean height) based on AFM scans (1×1 µm) of five areas of the same sample of MPA capped CuNPs on Si using two different tips. The data show the variation of the mean particle height when the tip is changed in-between scans.

Mean particle height (nm)<br><br>**Tip 1**	Mean particle height (nm)<br><br>**Tip 2**
10.8	9.4
10.8	9.9
10.2	8.9
11.1	8.3

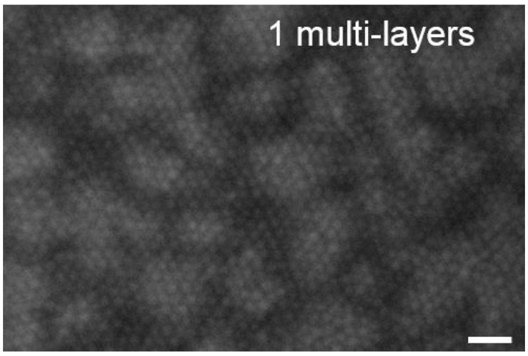

Supplementary Figure 6| Scanning electron microscopy image of a dense multi-layer oleylamine capped CuNP film on Si

Scale bar 200 nm.

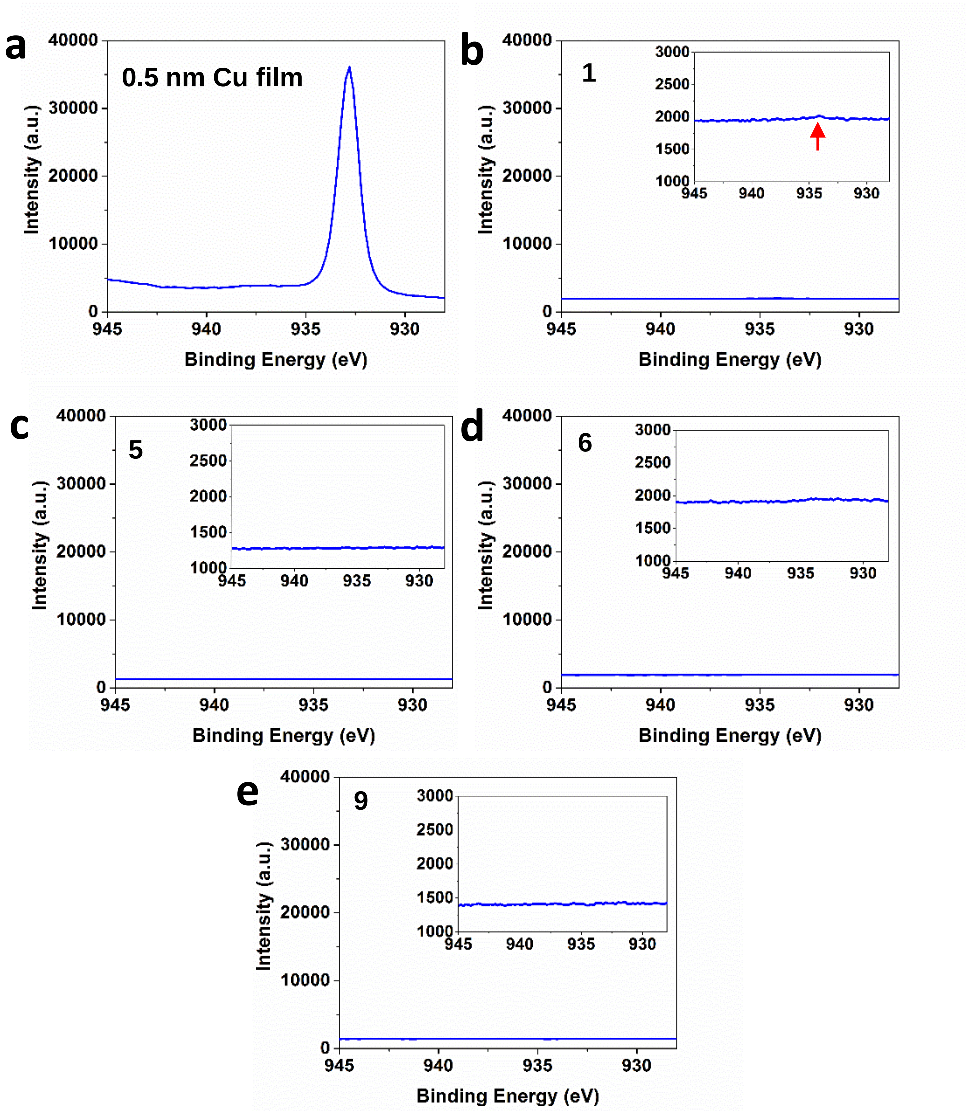

Supplementary Figure 7| High resolution XPS (Cu $2p_{3/2}$ region) of a thick region of solvent residue before and after 24 hours ligand exchange with ligands 5, 6 and 9

(a) 0.5 nm of Cu on Si wafer prepared by thermal evaporation. **(b-e)** Analysis of residue from 1 ml (from a total of 5.7 ml) of dried solvent (prepared by drop casting onto 1 cm^2 clean silicon substrates) after soaking a multilayer of oleylamine capped CuNPs on Si (Supplementary Figure 7) for 24 hours in: **(b)** ethanol only (1); **(c)** 6-mercaptohexanoic acid (5); **(d)** 1-octanethiol (6); **(e)** MPA (9). The red arrow in **(b)** draws attention to an extremely weak Cu peak from the residue from oleylamine capped CuNPs soaked in ethanol without thiol.

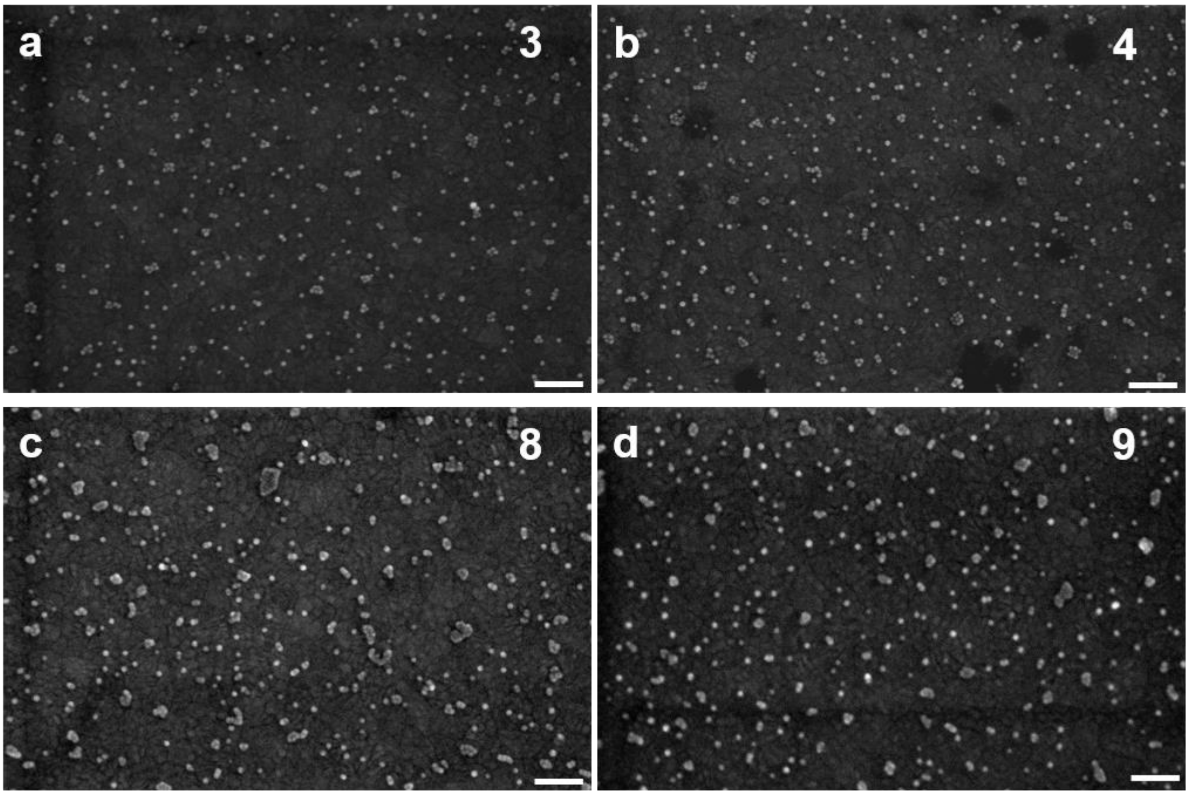

Supplementary Figure 8| CuNP density after the ligand exchange process

SEM images of CuNPs capped with ligands 3 **(a)**, 4 **(b)**, 8 **(c)** and 9 **(d)** supported on ITO glass. All images were captured at the same magnification of ×47150 for comparison, and the scale bar in all cases is 200 nm. SEM imaging of thiol treated samples was performed to test for the possibility of etching of CuNPs during ligand exchange. From the images there is no obvious reduction in the NP density with reference to oleylamine capped sample 1 (as synthesised CuNPs – Fig. 1c) when a similar area is imaged with the same magnification. These data support the conclusion that CuNPs are not etched when exposed to concentrated thiol solutions (i.e. 5×10^{-2} M) for prolonged periods of time (i.e. 24 hrs).

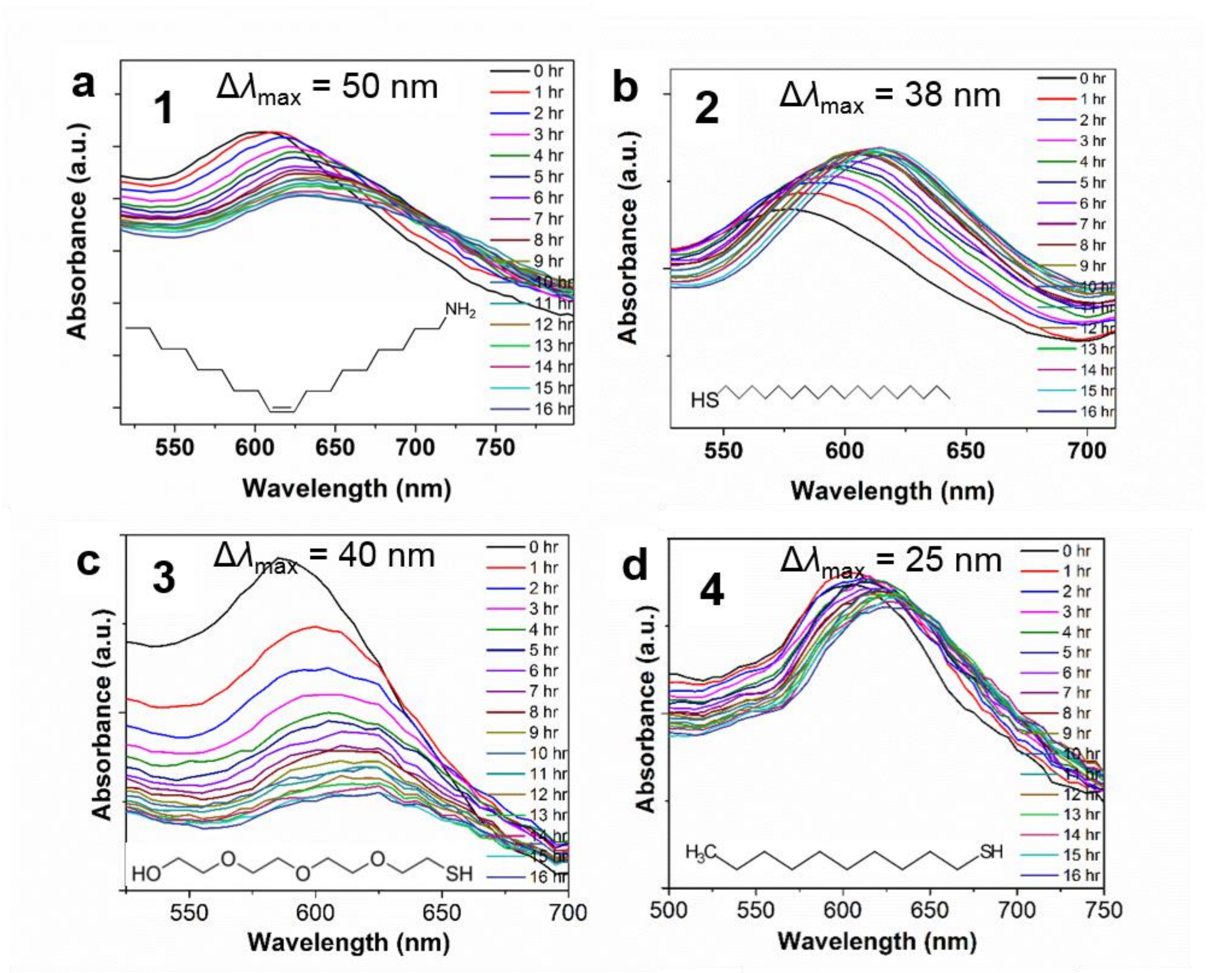
a
1
Δλmax = 50 nm
NH2
Absorbance (a.u.)
Wavelength (nm)
b
2
Δλmax = 38 nm
HS
c
3
Δλmax = 40 nm
HO O O O SH
d
4
Δλmax = 25 nm
H3C SH
0 hr
1 hr
2 hr
3 hr
4 hr
5 hr
6 hr
7 hr
8 hr
9 hr
10 hr
11 hr
12 hr
13 hr
14 hr
15 hr
16 hr

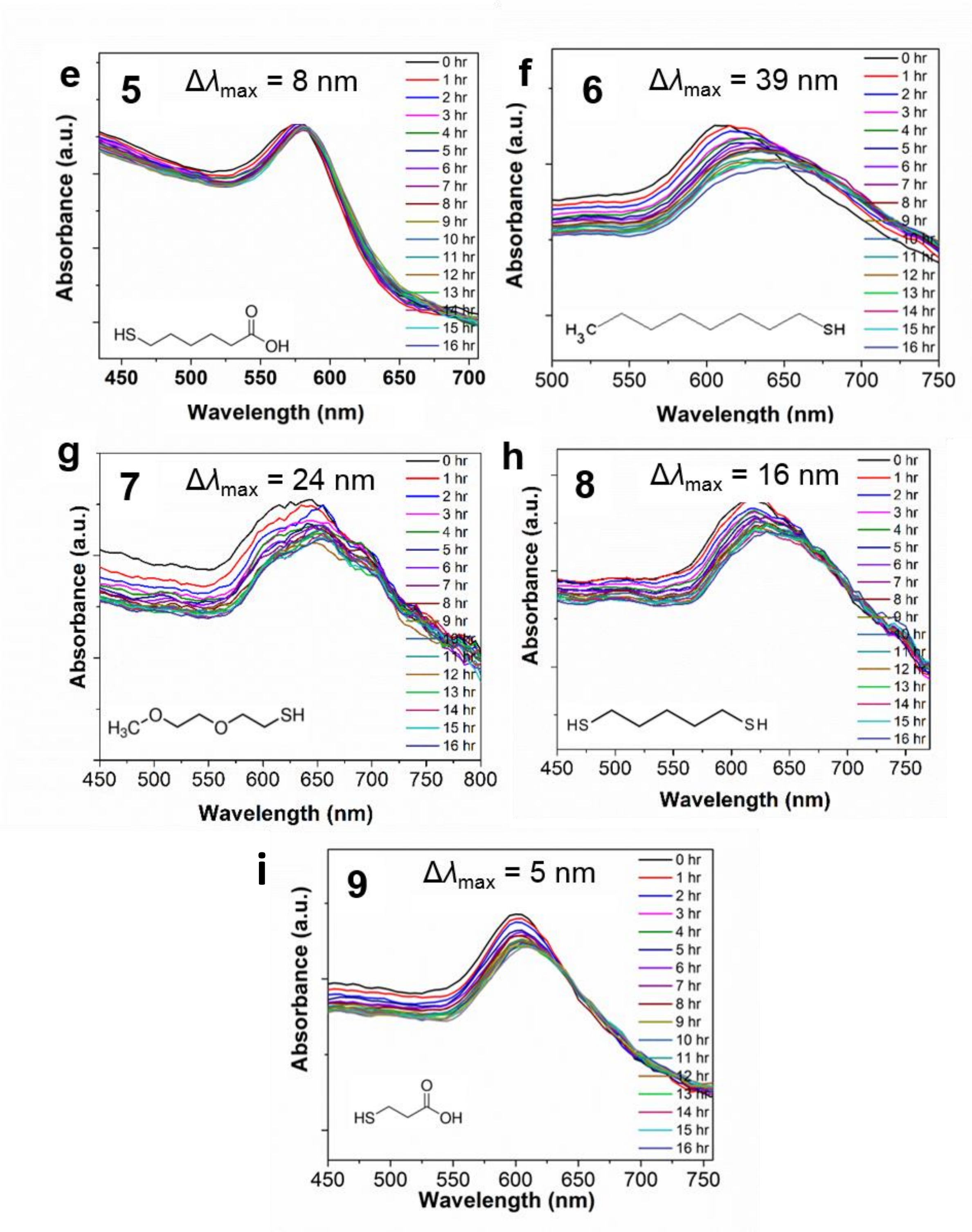

Supplementary Figure 9| Analysis of LSPR λ_{max} shift

UV-Vis extinction spectra of sub-monolayers of **(a)** 1, **(b)** 2, **(c)** 3, **(d)** 4, **(e)** 5, **(f)** 6, **(g)** 7, **(h)** 8 and **(i)** 9 on glass, monitored in ambient air over 16 hours.

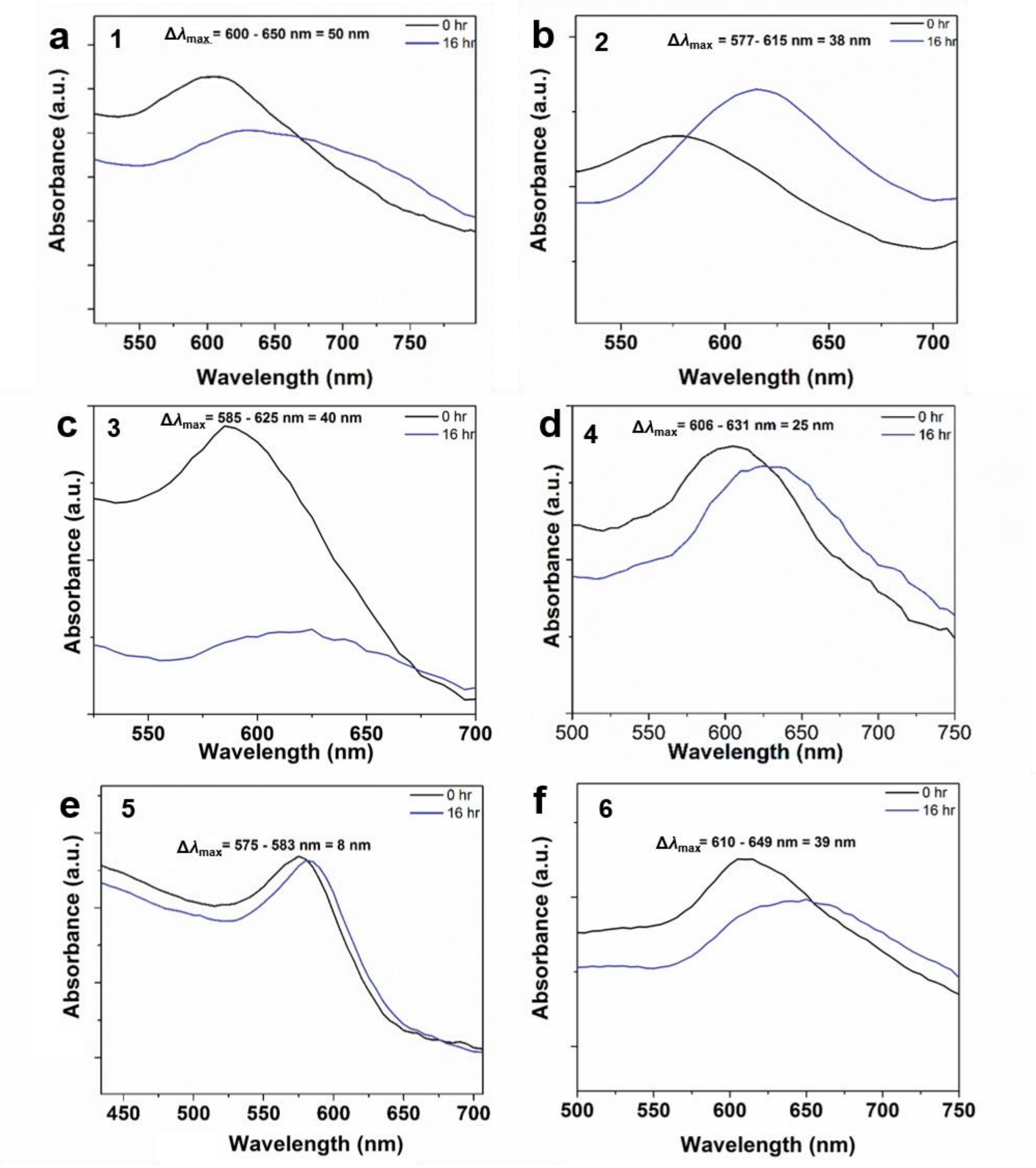
a
1
$\Delta\lambda_{max}$ = 600 - 650 nm = 50 nm
0 hr
16 hr
Absorbance (a.u.)
550 600 650 700 750
Wavelength (nm)
b
2
$\Delta\lambda_{max}$ = 577- 615 nm = 38 nm
0 hr
16 hr
Absorbance (a.u.)
550 600 650 700
Wavelength (nm)
c
3
$\Delta\lambda_{max}$ = 585 - 625 nm = 40 nm
0 hr
16 hr
Absorbance (a.u.)
550 600 650 700
Wavelength (nm)
d
4
$\Delta\lambda_{max}$ = 606 - 631 nm = 25 nm
0 hr
16 hr
Absorbance (a.u.)
500 550 600 650 700 750
Wavelength (nm)
e
5
$\Delta\lambda_{max}$ = 575 - 583 nm = 8 nm
0 hr
16 hr
Absorbance (a.u.)
450 500 550 600 650 700
Wavelength (nm)
f
6
$\Delta\lambda_{max}$ = 610 - 649 nm = 39 nm
0 hr
16 hr
Absorbance (a.u.)
500 550 600 650 700 750
Wavelength (nm)

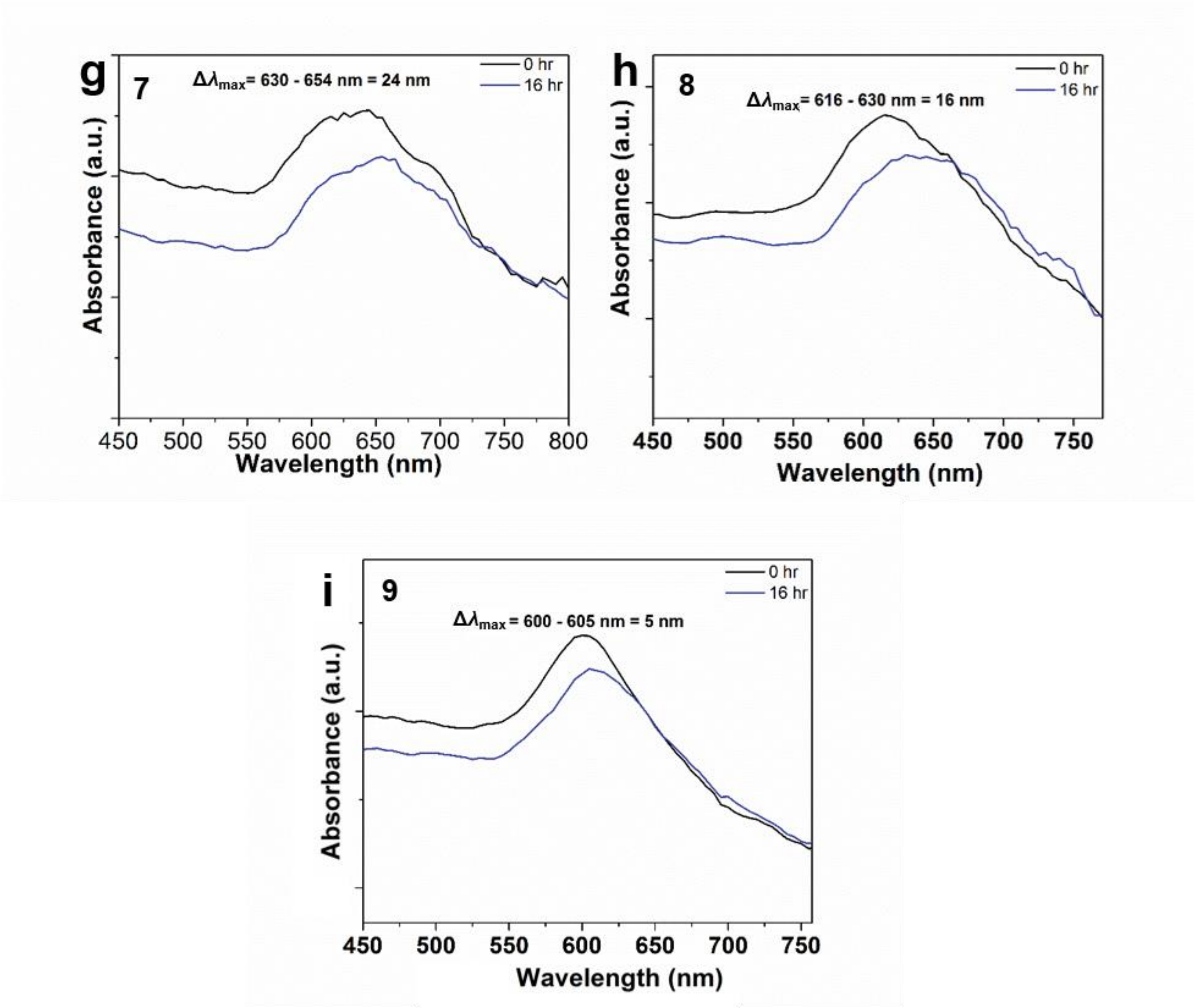

Supplementary Figure 10| Simplified version of Supplementary Figure 9

Analysis of LSPR λ_{max} shift and change in shape. UV-Vis extinction spectra of monolayers of **(a)** 1, **(b)** 2, **(c)** 3, **(d)** 4, **(e)** 5, **(f)** 6, **(g)** 7, **(h)** 8 and **(i)** 9 on glass, monitored in air at time zero (black) and after 16 hours (blue), from which it is evident that the change in shape of the LSPR peak is most pronounced for the least stable CuNPs.

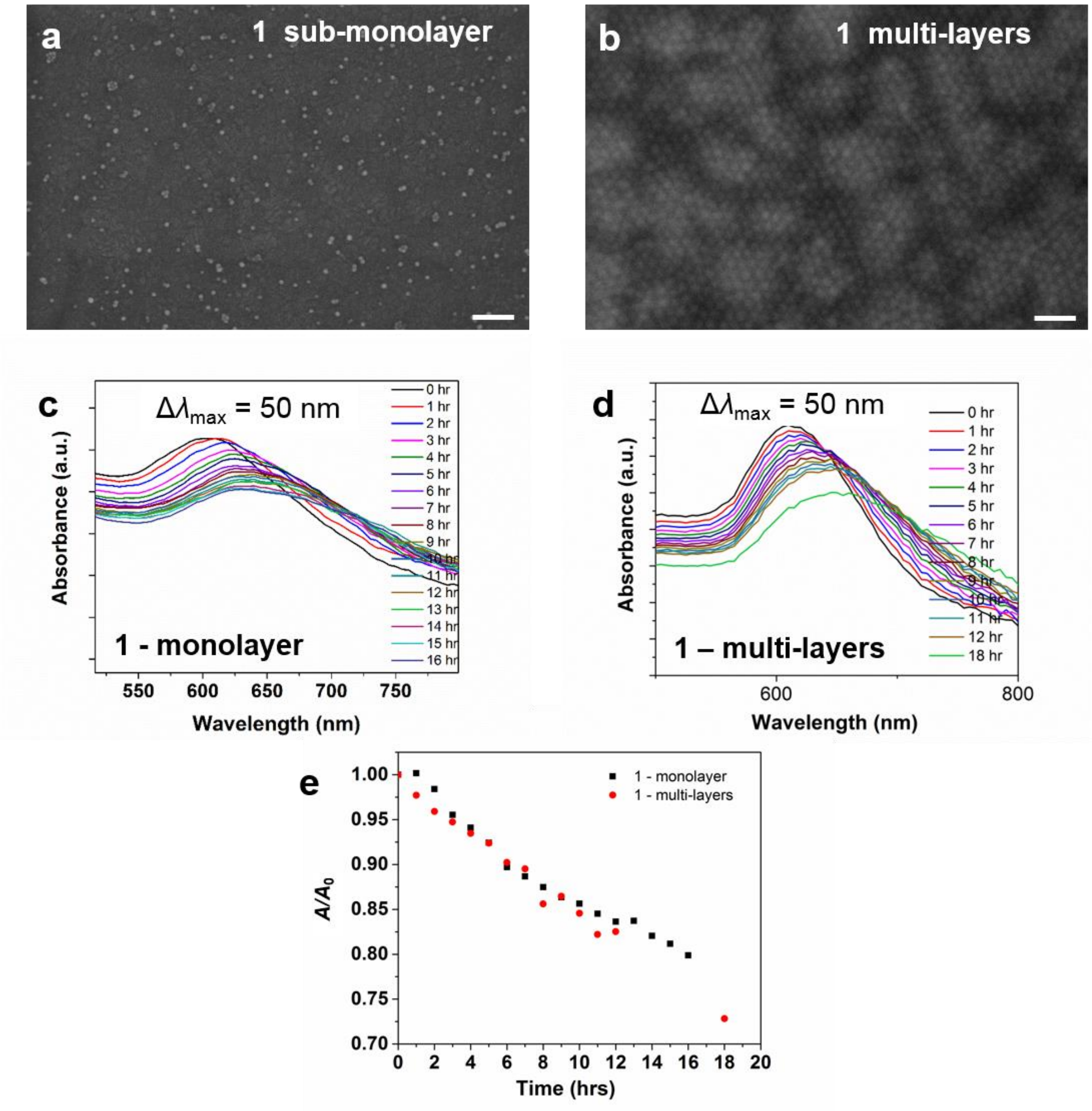

Supplementary Figure 11| Monitoring oxidation of CuNP films using extinction spectroscopy

SEM images of a sub-monolayer of oleylamine capped CuNPs **(a)** and a multilayer film of the same **(b)**. Scale bar 400 nm. Evolution of the extinction spectrum of a sub-monolayer **(c)** and multi-layer film **(d)** over 16-18 hrs in air. **(e)** LSPR peak degradation (A/A_0) for sub-monolayer and multilayers films of oleylamine capped CuNPs.

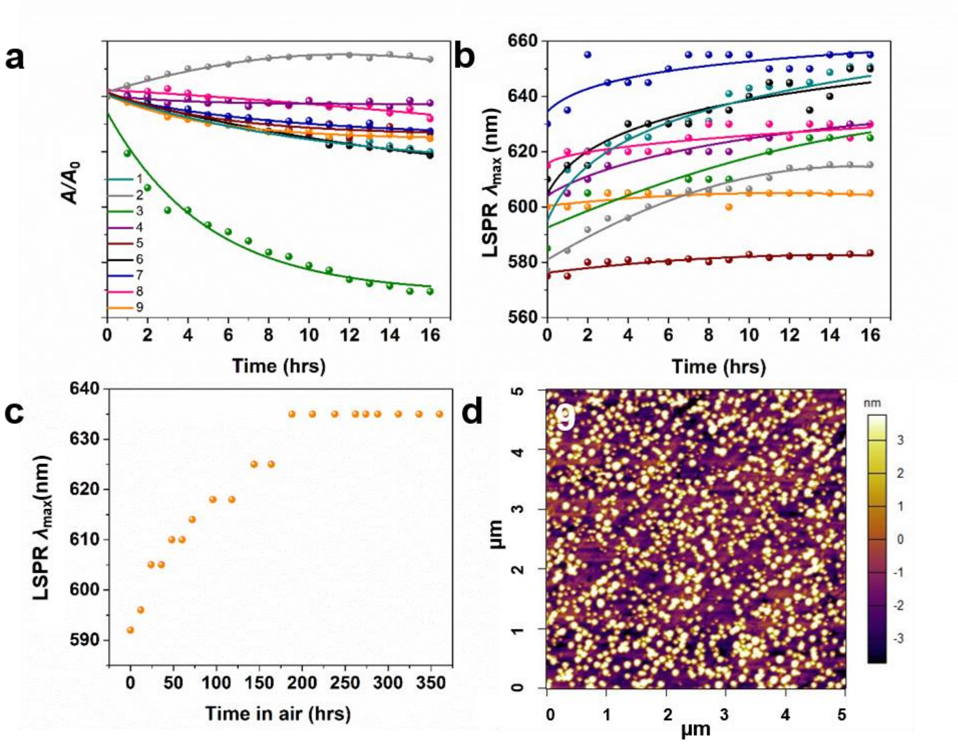

Supplementary Figure 12| Monitoring air-stability of thiol capped CuNPs

Plots of **(a)** A/A_0 vs time (where A is the LSPR absorption at various time intervals and A_0 is the absorption at time 0.) and **(b)** LSPR λ_{max} *vs* time for various ligand treated CuNP sub-monolayers showing the rate of degradation when exposed to air for 16 hrs. The change in A/A_0 shows an opposite trend for ligand 2, as compared with the rest of the ligands which exhibit a decrease of absorption intensity with time. **(c)** LSPR λ_{max} *vs* time for CuNPs capped with ligand (9) exposed to air over 15 days. **(d)** AFM image of ligand exchanged (9) sub-monolayer of CuNPs on glass used for UV-Vis measurements.

Supplementary Note 1| Literature reporting rate of oxidation of CuNPs in air

Barriere *et al.*[5] have shown that ~ 8.4 nm diameter CuNPs capped with hexadecylamine/diamine suspended in toluene exhibit a LSPR λ_{max} red-shift of ~31 nm after 24 hours. Similarly Rice *et al.*[6] have reported that ~11.7 nm CuNPs capped with oleic acid/trioctylamine and suspended in toluene exhibit a LSPR λ_{max} red-shift of ~30 nm during the first 20 min of air exposure and ~145 nm within 10 hrs. Kanninen *et al.*[7] have shown that the LSPR of ~3.3 nm CuNPs capped with 1-octadecanethiol (suspended in lauric acid/toluene) shifts by about 20-30 nm within 30 min of air exposure. For CuNPs (5 nm)

embedded within monolithic porous silica, Liu *et al.*[8] have reported a red shift of 6 nm of the LSPR band within 4 hrs of air exposure, 12 nm within 24 hrs and complete oxidation within 3 days.

Supplementary Table 3| Cu:N and Cu:S atomic ratios for CuNPs capped with ligands (1)-(9) calculated from high resolution Cu $2p_{3/2}$, N 1s and S 2p XPS core level spectra The data is presented in descending order of stability towards oxidation in air. Those data sets grouped with the same shade of grey background have comparable stability based on the analysis of the data presented in Figure 4 of the main manuscript.

Ligand	Cu:N	Cu:S
Oleylamine (1)	6.65:1	
2-{2-[2-(2-mercaptoethoxy)ethoxy]ethoxy}ethanol (3)		9.33:1
1-Octanethiol (6)		7.56:1
1-Octadecanethiol (2)		2.12:1
1-Decanethiol (4)		3.46:1
2-(2-methoxyethoxy)ethanethiol (7)		4.21:1
1,2-pentanedithiol (8)		1.5:1<br>0.75:1(Cu/2S)
6-mercaptohexanoic acid (5)		5.52:1
MPA (9)		1.49:1

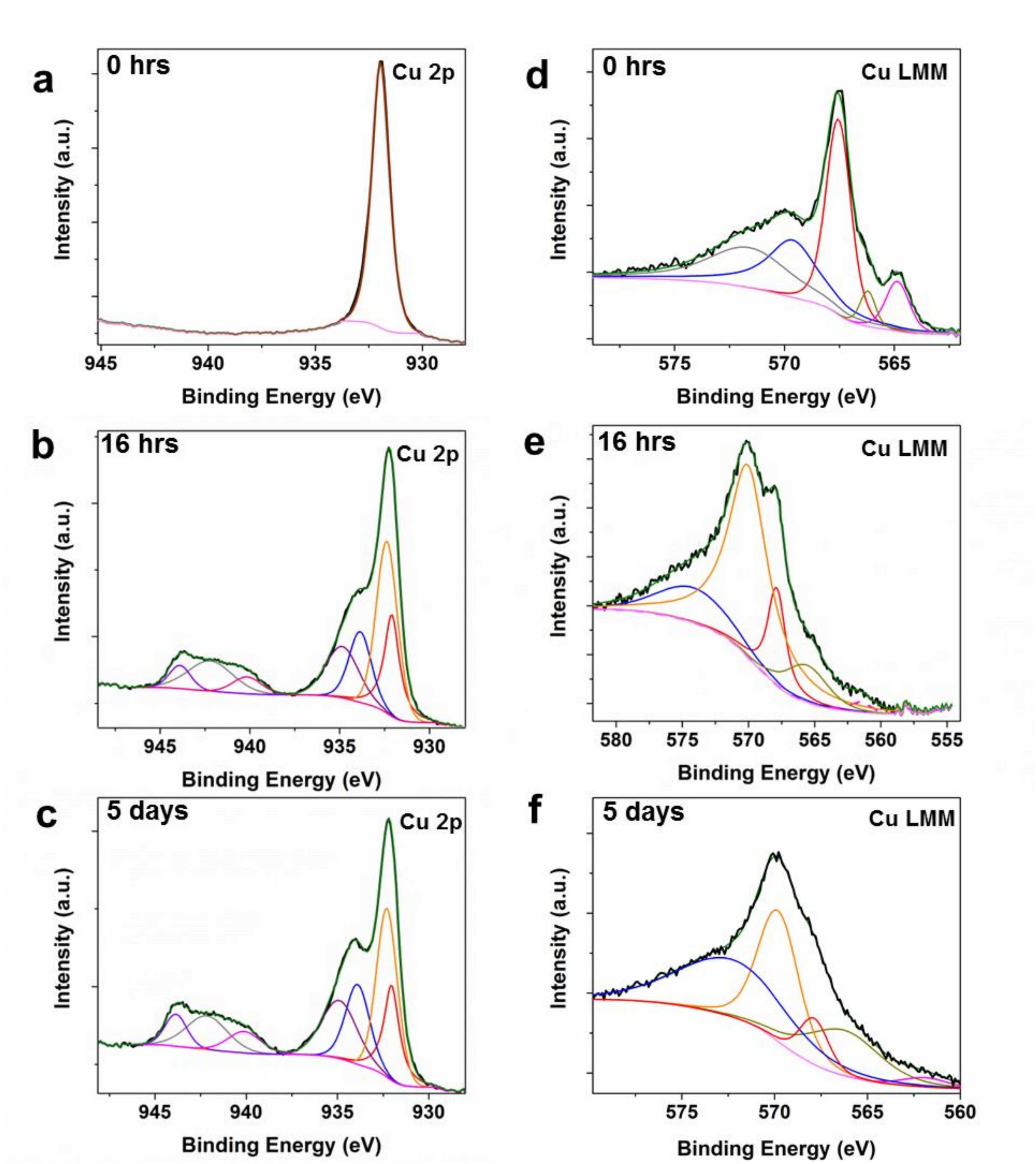

Supplementary Figure 13| Oxidation of ligand capped CuNPs in air

XPS core level Cu $2p_{3/2}$ spectra **(a-c)** and Cu LMM Auger spectra **(d-f)** of a sub-monolayer of MPA capped CuNPs (9) on Au coated silicon. The spectra correspond to samples exposed to ambient air for 0 hrs, 16 hrs and 5 days.

Supplementary Note 2| HRXPS analysis of Cu oxide thickness

The XPS sampling depth for $Cu2p_{3/2}$ electrons emitted from Cu_2O/CuO is ~3-3.5 nm (inelastic electron mean free paths calculated from the TPP-2M QUASES-IMFP-TPP2M Ver. 3.0)[9,10], hence the reason for enhanced intensities of Cu_2O/CuO peaks and less pronounced intensities for Cu^o .

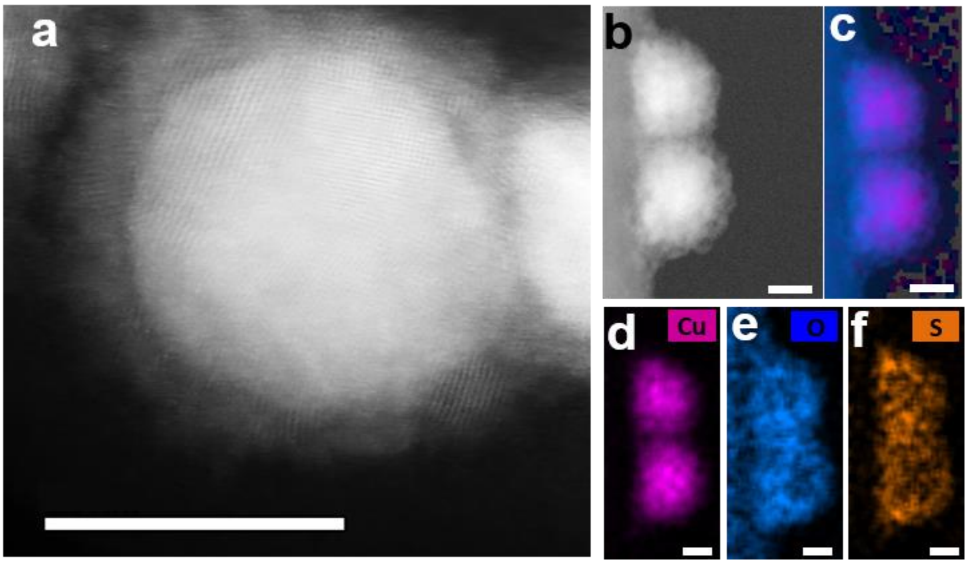

Supplementary Figure 14| Copper oxide core shell structure

(a) ADF-STEM image of a CuNP capped with MPA that has been exposed to air for 10 days. Scale bar 10 nm. **(b-f)** EDX elemental mapping of similar NPs displaying the core shell made up of copper oxide. Scale bar 5 nm.

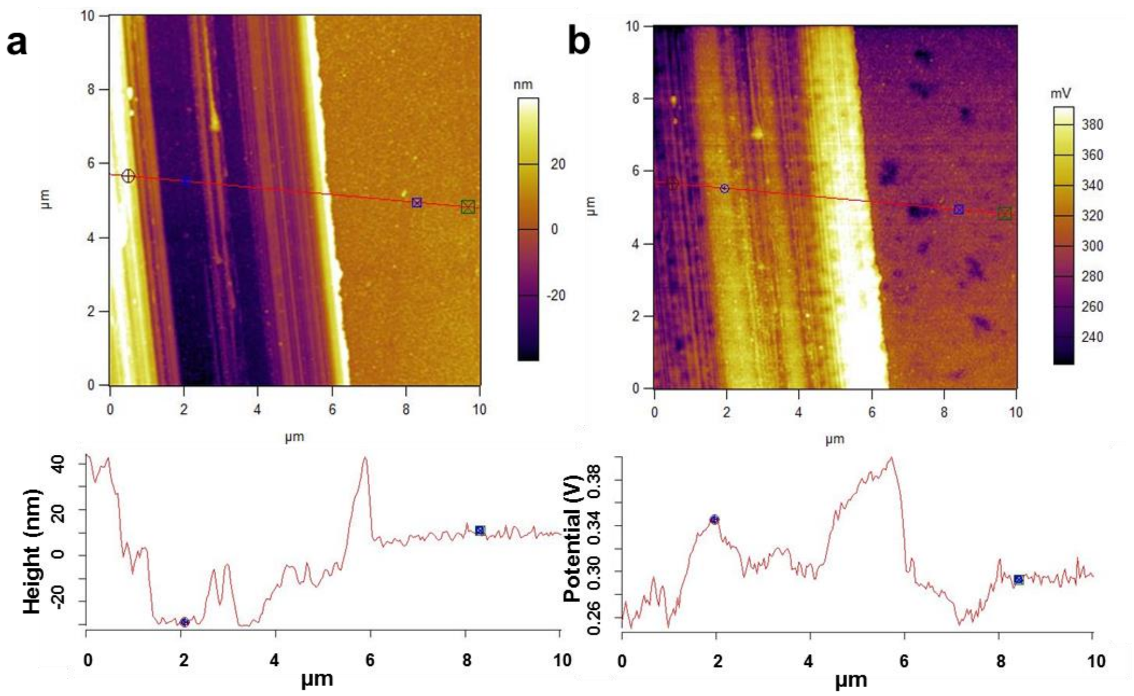

Supplementary Figure 15| KPFM analysis of Au on Al

AFM height image **(a)** and the corresponding KPFM potential image **(b)** of a 38 nm thick film of Au thermally evaporated onto 30 nm of Al. The underlying substrate is MPTMS functionalised Si. The Au layer was scored through, to expose the Al film underneath and a cross-section taken across the score. An increased contact potential on the Al surface with respect to the Au layer is observed.

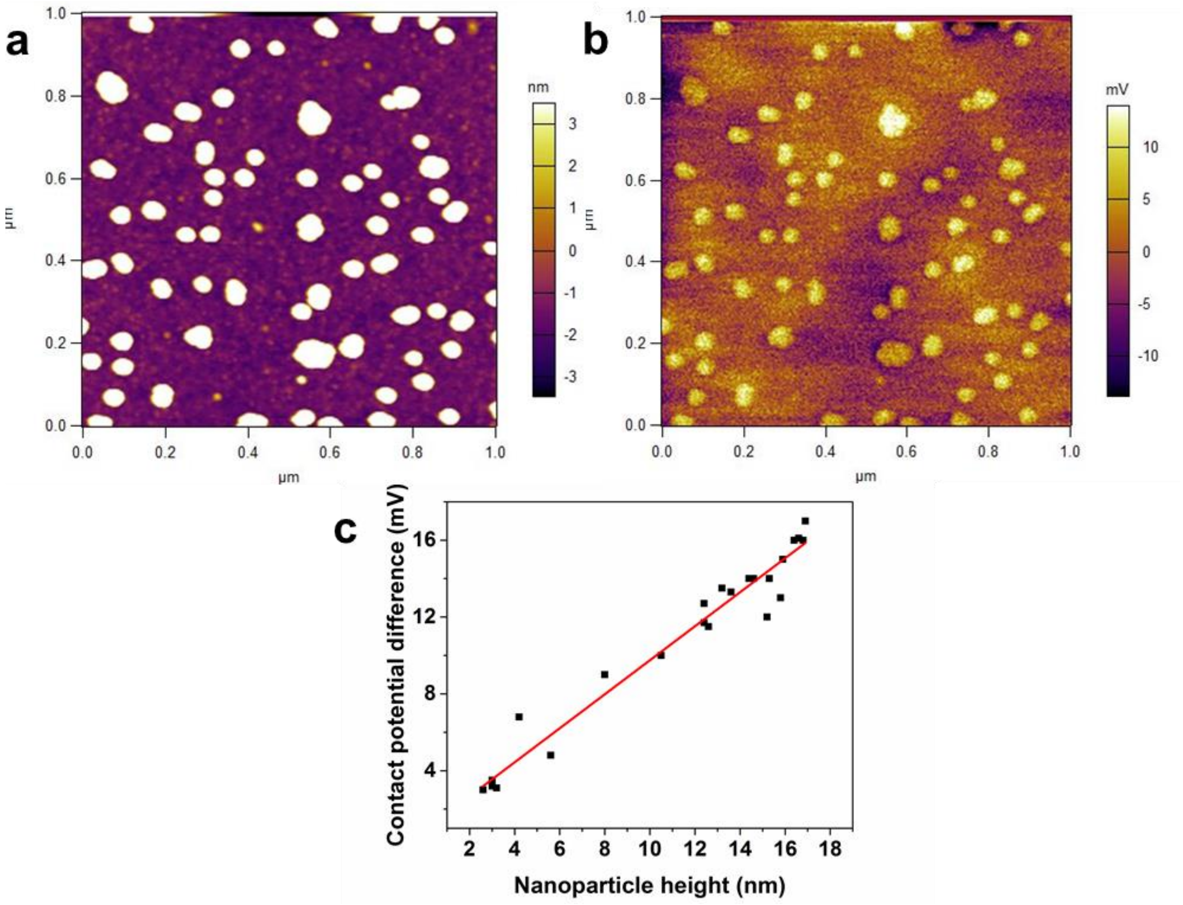

Supplementary Figure 16| KPFM analysis of an additional sample of MPA capped Cu NPs

This data corresponds to a repetition of the experiment shown in Figure 7 of the manuscript. **(a)** AFM height image of MPA capped CuNPs on n-Si. The error in CuNP height measurement for all data points is estimated to be ± 0.7 nm. **(b)** The corresponding contact potential difference image. **(c)** Contact potential difference *vs*. NP height for MPA capped CuNPs on n-Si. The same linear increase in the contact potential difference with CuNP height is observed as in the case of Figure 7 of the manuscript.

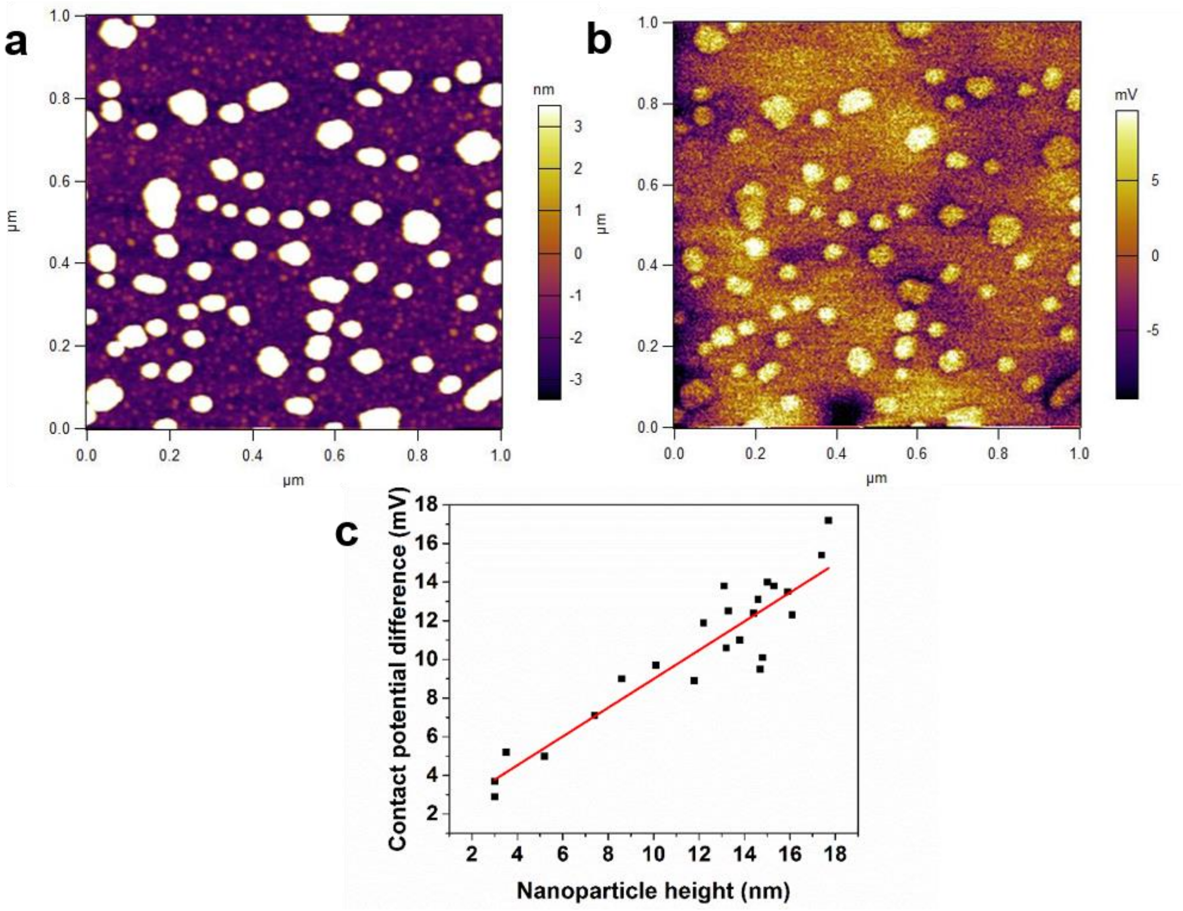

Supplementary Figure 17| KPFM analysis of oleylamine capped CuNPs on Si

(a) AFM height image of oleylamine capped CuNPs on n-Si. The error in CuNP height measurement for all data points is estimated to be ± 0.7 nm. **(b)** The corresponding contact potential difference image. **(c)** Contact potential difference *vs.* NP height for oleylamine capped CuNPs on n-Si. The dielectric environment is different to the case of MPA capped CuNPs, since the NPs are capped with the long chain, hydrophobic ligand oleylamine. However, similar to the case with MPA capped CuNPs, a linear increase in the contact potential difference of similar magnitude with CuNP height is observed.

Supplementary References

1 Ghahremaninezhad, A., Dixon, D. G. & Asselin, E. Electrochemical and XPS analysis of chalcopyrite ($CuFeS_2$) dissolution in sulfuric acid solution. *Electrochim. Acta* **87**, 97-112 (2013).

2 Platzman, I., Brener, R., Haick, H. & Tannenbaum, R. Oxidation of polycrystalline copper thin films at ambient conditions. *J. Phys. Chem. C* **112**, 1101-1108 (2008).

3 Alonso, C., López, M. F., Gutiérrez, A. & Escudero, M. L. X-ray photoelectron spectroscopy study of thiols adsorbed on Pt(111) with and without the presence of a copper monolayer. *Surf. Interface Anal.* **30**, 359-363 (2000).

4 Vericat, C., Vela, M. E., Benitez, G., Carro, P. & Salvarezza, R. C. Self-assembled monolayers of thiols and dithiols on gold: new challenges for a well-known system. *Chem. Soc. Rev.* **39**, 1805-1834 (2010).

5 Barriere, C. *et al.* Ligand effects on the air stability of copper nanoparticles obtained from organometallic synthesis. *J. Mater. Chem.* **22**, 2279-2285 (2012).

6 Kanninen, P., Johans, C., Merta, J. & Kontturi, K. Influence of ligand structure on the stability and oxidation of copper nanoparticles. *J. Colloid Interface Sci.* **318**, 88-95 (2008).

7 Rice, K. P., Walker, E. J., Stoykovich, M. P. & Saunders, A. E. Solvent-dependent surface plasmon response and oxidation of copper nanocrystals. *J. Phys. Chem. C* **115**, 1793-1799 (2011).

8 Liu, X., Cai, W. & Bi, H. Optical absorption of copper nanoparticles dispersed within pores of monolithic mesoporous silica. *J. Mater. Res.* **17**, 1125-1128.

9 Tanuma, S., Powell, C. J. & Penn, D. R. Calculations of electron inelastic mean free paths. *Surf. Interface Anal.* **37**, 1-14 (2005).

10 Tanuma, S., Powell, C. J. & Penn, D. R. Calculations of electron inelastic mean free paths. V. Data for 14 organic compounds over the 50–2000 eV range. *Surf. Interface Anal.* **21**, 165-176 (1994).
